# A New Test for Urinary Sugar

**Published:** 1916-04

**Authors:** 


**A New Test for Urinary Sugar.** The difficulties associated with the performance of the ordinary tests for sugar in the urine, of which Fehling's is by far the most commonly employed, are well known, so that the invention of a new diagnostic reagent is a matter of considerable importance. In the Biochemical Journal for March, Dr. William Cramer describes a method of employing the reduction of a weakly alkaline solution of mercuric oxide to metallic mercury in the presence of suitable sugars, which he considers to have special advantages as a test. The reagent is prepared by dissolving 0.4 gr. of mercuric oxide, red or yellow, with 6 gr. of potassium iodine in 100 c.c. of water, and adjusting the alkalinity of the solution so that 10 c.c. are neutralized to phenol-phthalein by 2.5 c.c. of decinormal acid. The resulting solution is colorless in the cold and turns slightly yellow on boiling. To test for sugar in urine 3 c.c. of the reagent are heated to boiling; then 0.3 c.c. of urine is added and the mixture again boiled. On removing the test-tube from the flame, the mixture darkens if sugar be present, and a deposit of black metallic mercury gradually settles to the bottom. Not only glucose, but lactose, maltose, xylose, and arabinose give the reaction, while cane sugar does not. The sensitiveness of the test fluid can be varied by increasing or diminishing its degree of alkalinity, and the point fixed, as stated above, corresponds to a degree which gives a faint reaction with normal urine, which is known to contain about 0.1 per cent of sugar. If the reagent is made more alkaline and thus more sensitive, it may give a precipitate with creatinin and other organic substances. Dr. Cramer finds that the "2.5 standard" solution gives a definite reaction with small amounts of sugar in the urine, which only produce doubtful results when tested with Fehling's fluid. He suggests as an indication of the degree of the reaction the possibility of reading print through the solution when boiled in an ordinary test-tube 30 seconds after this is removed from the flame, a few drops of acetic acid being first added to remove any possible turbidity due to phosphates. Small quantities of sugar can thus be recognized and roughly estimated. Ammoniacal urine must not be used for the mercuric test any more than in performing Fehling's reaction.—Med. Fortnightly.

